# Ethyl (2*R*,3*S*)-2-benzoyl-3-(4-bromo­phen­yl)-4-nitro­butano­ate

**DOI:** 10.1107/S1600536812002073

**Published:** 2012-01-21

**Authors:** Yifeng Wang, Ke Wang, Zhaobo Li, Danqian Xu

**Affiliations:** a Catalytic Hydrogenation Research Center, Zhejiang University of Technology, Hangzhou 310014, People’s Republic of China; b Hangzhou Minsheng Pharmaceutical Group Co. Ltd, Hangzhou, People’s Republic of China

## Abstract

The title compoud, C_19_H_18_BrNO_5_, was synthesized by an organocatalytic reaction. The aymmetric unit contains two independent mol­ecules, in each of which the carbon between the two carbonyl groups adopts an *R* configuration, while the adjacent C atom has an *S* configuration. The dihedral angle between the two benzene rings is different in the two mol­ecules [11.64 (3) and 58.96 (4)°].

## Related literature

For the asymmetric synthesis of the title compound, see: Bae *et al.* (2011 ▶); Malerich *et al.* (2008 ▶).
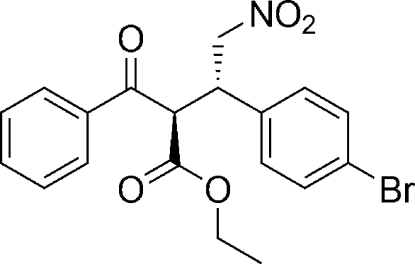


## Experimental

### 

#### Crystal data

C_19_H_18_BrNO_5_
*M* *_r_* = 420.25
Monoclinic, 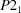

*a* = 5.7558 (3) Å
*b* = 21.6262 (9) Å
*c* = 15.1337 (7) Å
β = 93.720 (1)°
*V* = 1879.81 (15) Å^3^
*Z* = 4
Mo *K*α radiation
μ = 2.22 mm^−1^
*T* = 296 K
0.48 × 0.30 × 0.27 mm

#### Data collection

Rigaku R-AXIS RAPID/ZJUG diffractometer
Absorption correction: multi-scan (*ABSCOR*; Higashi, 1995 ▶) *T*_min_ = 0.346, *T*_max_ = 0.549
16125 measured reflections
7326 independent reflections
4316 reflections with *I* > 2σ(*I*)
*R*_int_ = 0.043

#### Refinement

*R*[*F* ^2^ > 2σ(*F* ^2^)] = 0.046
*wR*(*F* ^2^) = 0.127
*S* = 1.00
7326 reflections
469 parameters
27 restraints
H-atom parameters constrained
Δρ_max_ = 0.42 e Å^−3^
Δρ_min_ = −0.54 e Å^−3^
Absolute structure: Flack (1983 ▶), 3541 Friedel pairs
Flack parameter: −0.013 (9)

### 

Data collection: *PROCESS-AUTO* (Rigaku, 2007 ▶); cell refinement: *PROCESS-AUTO*; data reduction: *CrystalClear* (Rigaku, 2007 ▶); program(s) used to solve structure: *SHELXS97* (Sheldrick, 2008 ▶); program(s) used to refine structure: *SHELXL97* (Sheldrick, 2008 ▶); molecular graphics: *ORTEP-3 for Windows* (Farrugia, 1997 ▶); software used to prepare material for publication: *WinGX* (Farrugia, 1999 ▶).

## Supplementary Material

Crystal structure: contains datablock(s) global, I. DOI: 10.1107/S1600536812002073/bt5738sup1.cif

Structure factors: contains datablock(s) I. DOI: 10.1107/S1600536812002073/bt5738Isup2.hkl

Supplementary material file. DOI: 10.1107/S1600536812002073/bt5738Isup3.cml

Additional supplementary materials:  crystallographic information; 3D view; checkCIF report

## Figures and Tables

**Fig. 1.**
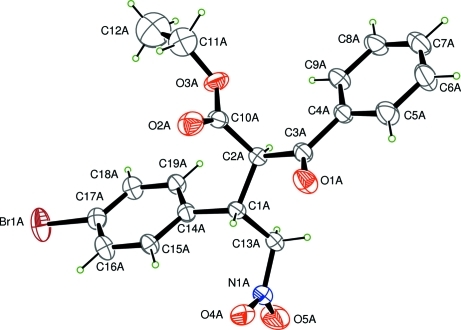
One of the structure (a) of the title compound. Displacement ellipsoids are drawn at the 50% probability level.

**Fig. 2.**
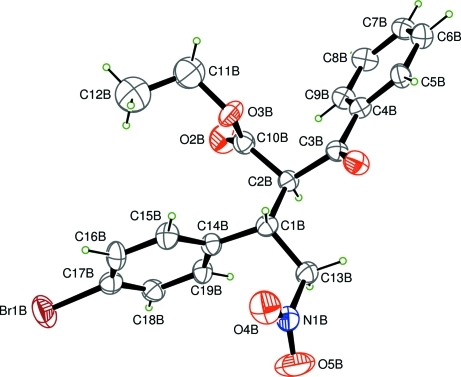
One of the structure (b) of the title compound. Displacement ellipsoids are drawn at the 50% probability level.

**Fig. 3.**
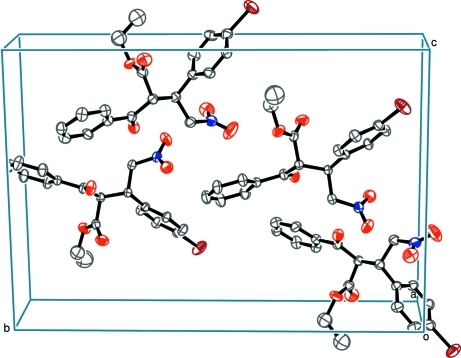
The molecular packing of the title compound.

## supplementary crystallographic information

### Comment

Organocatalytic Michael addition of 1,3-dicarbonyl compounds to nitroolefins has recently been extensively explored because of its offering an extremely effective way to synthesize a variety of useful chiral functionalized organic molecules. The title compound, which could be readily synthesized through organocatalytic Michael reaction of ethyl 3-oxo-3-phenylpropanoate to (*E*)-1-bromo-4-(2-nitrovinyl)benzene, could act as intermediate in organic and natural product synthesis. In this article, the crystal structure of the title compoud (2*R*,3*S*)-ethyl 2-benzoyl-3-(4-bromophenyl)-4-nitrobutanoate was determined (Fig. 1). The aymmetric unit has two independent molecules, in each of which the carbon between the two carbonyl groups adopts an *R* conformation, while the adjacent carbon atom is *S* conformation. In the two molecules, the dihedral angle between the two benzene rings differs [11.64 (3) and 58.96 (4)°].

### Experimental

To a solution of (*E*)-1-bromo-4-(2-nitrovinyl)benzene (1 mmol) and ethyl 3-oxo-3-phenylpropanoate (1 mmol) in 1,4-Dioxane (3 ml) was added 3-((1*S*)-(6-methoxyquinolin-4-yl) (8-vinylquinuclidin-2-yl)methylamino)-4- ((*S*)-1-phenylethylamino)cyclobut -3-ene-1,2-dione(0.025 mmol) as catalyst, and the mixture was stirred at room temperature for 12 h (monitored by TLC). Then the solvent was distilled under vacuum, and the residue was purified by flash column chromatography (silica gel, Hex/AcOEt, *v*/*v*, 3:1) giving the title compound. Single crystals were obtained by slow evaporation of a CH_2_Cl_2_ and *i*PrOH solution.

### Refinement

The carbon atoms of the ethyl groups (C11A C12A C11B C12B) were restrained to be approximately isotropic. And the C—C bond lengths of C11A—C12A and C11B—C12B were restrained to a target value of 1.535 Å, with a standard deviation of 0.001. H atoms were placed in calculated position with C—H=0.98 Å (*sp*), C—H=0.97 Å (sp^2^), C—H=0.96 Å (sp^3^), C—H=0.93 Å (aromatic) and with *U*_iso_(H)=1.2*U*_eq_ of the carrier atoms.

### Crystal data

**Table:**

C_19_H_18_BrNO_5_	*F*(000) = 856
*M**_r_* = 420.25	*D*_x_ = 1.485 Mg m^−^^3^
Monoclinic, *P*2_1_	Mo *K*α radiation, λ = 0.71073 Å
Hall symbol: P 2yb	Cell parameters from 11156 reflections
*a* = 5.7558 (3) Å	θ = 3.1–27.4°
*b* = 21.6262 (9) Å	µ = 2.22 mm^−^^1^
*c* = 15.1337 (7) Å	*T* = 296 K
β = 93.720 (1)°	Needle, colorless
*V* = 1879.81 (15) Å^3^	0.48 × 0.30 × 0.27 mm
*Z* = 4	

### Data collection

**Table:**

Rigaku R-AXIS RAPID/ZJUG diffractometer	7326 independent reflections
Radiation source: rotating anode	4316 reflections with *I* > 2σ(*I*)
graphite	*R*_int_ = 0.043
Detector resolution: 10.00 pixels mm^-1^	θ_max_ = 26.0°, θ_min_ = 3.1°
ω scans	*h* = −7→6
Absorption correction: multi-scan (*ABSCOR*; Higashi, 1995)	*k* = −26→26
*T*_min_ = 0.346, *T*_max_ = 0.549	*l* = −18→18
16125 measured reflections	

### Refinement

**Table:**

Refinement on *F*^2^	Secondary atom site location: difference Fourier map
Least-squares matrix: full	Hydrogen site location: inferred from neighbouring sites
*R*[*F*^2^ > 2σ(*F*^2^)] = 0.046	H-atom parameters constrained
*wR*(*F*^2^) = 0.127	*w* = 1/[σ^2^(*F*_o_^2^) + (0.0512*P*)^2^ + 0.3123*P*] where *P* = (*F*_o_^2^ + 2*F*_c_^2^)/3
*S* = 1.00	(Δ/σ)_max_ = 0.001
7326 reflections	Δρ_max_ = 0.42 e Å^−^^3^
469 parameters	Δρ_min_ = −0.54 e Å^−^^3^
27 restraints	Absolute structure: Flack (1983), 3541 Friedel pairs
Primary atom site location: structure-invariant direct methods	Flack parameter: −0.013 (9)

### Special details

**Table:**

Geometry. All e.s.d.'s (except the e.s.d. in the dihedral angle between two l.s. planes) are estimated using the full covariance matrix. The cell e.s.d.'s are taken into account individually in the estimation of e.s.d.'s in distances, angles and torsion angles; correlations between e.s.d.'s in cell parameters are only used when they are defined by crystal symmetry. An approximate (isotropic) treatment of cell e.s.d.'s is used for estimating e.s.d.'s involving l.s. planes.
Refinement. Refinement of *F*^2^ against ALL reflections. The weighted *R*-factor *wR* and goodness of fit *S* are based on *F*^2^, conventional *R*-factors *R* are based on *F*, with *F* set to zero for negative *F*^2^. The threshold expression of *F*^2^ > σ(*F*^2^) is used only for calculating *R*-factors(gt) *etc*. and is not relevant to the choice of reflections for refinement. *R*-factors based on *F*^2^ are statistically about twice as large as those based on *F*, and *R*- factors based on ALL data will be even larger.

### Fractional atomic coordinates and isotropic or equivalent isotropic displacement parameters (Å^2^)

**Table:**

	*x*	*y*	*z*	*U*_iso_*/*U*_eq_	
Br1A	1.29441 (11)	0.56418 (3)	0.17401 (5)	0.0918 (3)	
O1A	0.4552 (6)	0.81610 (17)	0.4794 (3)	0.0671 (11)	
O2A	0.6040 (7)	0.7964 (2)	0.2713 (3)	0.0736 (11)	
O3A	0.9340 (7)	0.85124 (18)	0.2842 (2)	0.0662 (10)	
O4A	0.6867 (9)	0.6324 (2)	0.5360 (3)	0.0795 (12)	
O5A	1.0065 (11)	0.6395 (2)	0.6147 (4)	0.1024 (17)	
N1A	0.8595 (10)	0.6601 (2)	0.5607 (3)	0.0626 (12)	
C1A	0.7975 (8)	0.7339 (2)	0.4326 (3)	0.0424 (11)	
H1A	0.6321	0.7227	0.4288	0.051*	
C2A	0.8168 (8)	0.8036 (2)	0.4127 (3)	0.0403 (10)	
H2A	0.9740	0.8179	0.4312	0.048*	
C3A	0.6384 (9)	0.8405 (2)	0.4625 (3)	0.0476 (12)	
C4A	0.6864 (9)	0.9065 (2)	0.4869 (3)	0.0466 (12)	
C5A	0.5307 (10)	0.9361 (3)	0.5379 (4)	0.0645 (15)	
H5A	0.4035	0.9145	0.5570	0.077*	
C6A	0.5620 (12)	0.9975 (3)	0.5609 (5)	0.0782 (19)	
H6A	0.4538	1.0178	0.5936	0.094*	
C7A	0.7557 (11)	1.0283 (3)	0.5348 (4)	0.0710 (17)	
H7A	0.7801	1.0693	0.5514	0.085*	
C8A	0.9117 (11)	0.9994 (3)	0.4848 (4)	0.0668 (17)	
H8A	1.0387	1.0214	0.4662	0.080*	
C9A	0.8838 (9)	0.9384 (2)	0.4617 (4)	0.0568 (14)	
H9A	0.9943	0.9185	0.4297	0.068*	
C10A	0.7691 (9)	0.8160 (2)	0.3148 (4)	0.0472 (12)	
C11A	0.9041 (14)	0.8701 (4)	0.1943 (5)	0.101 (2)	
H11A	0.8619	0.9135	0.1916	0.121*	
H11B	0.7782	0.8466	0.1647	0.121*	
C12A	1.1267 (16)	0.8601 (6)	0.1466 (8)	0.160 (4)	
H12A	1.1016	0.8728	0.0859	0.240*	
H12B	1.1682	0.8171	0.1490	0.240*	
H12C	1.2504	0.8842	0.1749	0.240*	
C13A	0.8929 (10)	0.7242 (2)	0.5278 (3)	0.0533 (13)	
H13A	1.0579	0.7338	0.5319	0.064*	
H13B	0.8167	0.7529	0.5658	0.064*	
C14A	0.9208 (8)	0.6937 (2)	0.3675 (3)	0.0428 (11)	
C15A	0.8224 (8)	0.6384 (2)	0.3383 (3)	0.0460 (12)	
H15A	0.6796	0.6267	0.3585	0.055*	
C16A	0.9288 (9)	0.5998 (3)	0.2802 (4)	0.0587 (14)	
H16A	0.8589	0.5627	0.2623	0.070*	
C17A	1.1408 (8)	0.6170 (3)	0.2490 (4)	0.0524 (13)	
C18A	1.2407 (9)	0.6726 (3)	0.2778 (4)	0.0575 (14)	
H18A	1.3830	0.6844	0.2574	0.069*	
C19A	1.1330 (8)	0.7107 (2)	0.3363 (3)	0.0485 (12)	
H19A	1.2030	0.7476	0.3547	0.058*	
Br1B	0.63666 (15)	0.41912 (3)	1.11381 (5)	0.0991 (3)	
O1B	0.5085 (6)	0.70722 (17)	0.6957 (2)	0.0607 (10)	
O2B	0.1843 (7)	0.6699 (2)	0.9555 (3)	0.0829 (13)	
O3B	0.5254 (7)	0.7107 (2)	0.9220 (2)	0.0708 (11)	
O4B	0.8589 (9)	0.5332 (2)	0.7336 (4)	0.0899 (15)	
O5B	0.6010 (12)	0.4713 (3)	0.6777 (5)	0.147 (3)	
N1B	0.6594 (11)	0.5201 (3)	0.7085 (3)	0.0722 (14)	
C1B	0.5169 (8)	0.6042 (2)	0.8032 (3)	0.0474 (12)	
H1B	0.6666	0.6255	0.8007	0.057*	
C2B	0.3228 (8)	0.6546 (2)	0.8097 (3)	0.0450 (11)	
H2B	0.1709	0.6351	0.7962	0.054*	
C3B	0.3503 (8)	0.7079 (2)	0.7448 (3)	0.0468 (12)	
C4B	0.1843 (9)	0.7606 (2)	0.7434 (3)	0.0498 (12)	
C5B	0.2286 (10)	0.8121 (3)	0.6921 (4)	0.0553 (14)	
H5B	0.3589	0.8128	0.6588	0.066*	
C6B	0.0800 (11)	0.8622 (3)	0.6902 (4)	0.0674 (16)	
H6B	0.1122	0.8967	0.6563	0.081*	
C7B	−0.1159 (10)	0.8617 (3)	0.7381 (4)	0.0611 (15)	
H7B	−0.2171	0.8953	0.7356	0.073*	
C8B	−0.1600 (10)	0.8112 (3)	0.7895 (4)	0.0667 (16)	
H8B	−0.2899	0.8110	0.8230	0.080*	
C9B	−0.0138 (9)	0.7608 (3)	0.7918 (4)	0.0569 (14)	
H9B	−0.0475	0.7266	0.8259	0.068*	
C10B	0.3312 (10)	0.6787 (3)	0.9041 (4)	0.0555 (13)	
C11B	0.5567 (14)	0.7394 (4)	1.0100 (5)	0.105 (2)	
H11C	0.4056	0.7479	1.0322	0.126*	
H11D	0.6385	0.7784	1.0055	0.126*	
C12B	0.6951 (17)	0.6968 (4)	1.0744 (6)	0.133 (3)	
H12D	0.7124	0.7160	1.1316	0.199*	
H12E	0.8460	0.6892	1.0531	0.199*	
H12F	0.6138	0.6582	1.0789	0.199*	
C13B	0.4719 (8)	0.5678 (3)	0.7169 (3)	0.0565 (13)	
H13C	0.3213	0.5477	0.7166	0.068*	
H13D	0.4697	0.5959	0.6669	0.068*	
C14B	0.5342 (7)	0.5612 (3)	0.8821 (3)	0.0459 (11)	
C15B	0.7338 (8)	0.5612 (3)	0.9380 (4)	0.0590 (13)	
H15B	0.8511	0.5896	0.9287	0.071*	
C16B	0.7630 (10)	0.5195 (3)	1.0080 (4)	0.0648 (16)	
H16B	0.8984	0.5200	1.0450	0.078*	
C17B	0.5928 (10)	0.4783 (3)	1.0216 (4)	0.0612 (15)	
C18B	0.3873 (10)	0.4772 (3)	0.9684 (4)	0.0584 (14)	
H18B	0.2699	0.4492	0.9792	0.070*	
C19B	0.3612 (9)	0.5190 (3)	0.8987 (4)	0.0546 (13)	
H19B	0.2246	0.5186	0.8624	0.066*	

### Atomic displacement parameters (Å^2^)

**Table:**

	*U*^11^	*U*^22^	*U*^33^	*U*^12^	*U*^13^	*U*^23^
Br1A	0.0684 (4)	0.1068 (6)	0.1006 (5)	0.0089 (4)	0.0095 (4)	−0.0550 (5)
O1A	0.058 (2)	0.051 (2)	0.095 (3)	−0.0120 (19)	0.029 (2)	−0.011 (2)
O2A	0.077 (3)	0.080 (3)	0.061 (3)	−0.012 (2)	−0.016 (2)	0.010 (2)
O3A	0.078 (3)	0.069 (3)	0.052 (2)	−0.009 (2)	0.0129 (19)	0.020 (2)
O4A	0.110 (3)	0.067 (3)	0.062 (3)	−0.026 (3)	0.016 (3)	0.007 (2)
O5A	0.144 (4)	0.063 (3)	0.096 (4)	0.020 (3)	−0.031 (3)	0.020 (3)
N1A	0.095 (4)	0.043 (3)	0.051 (3)	−0.006 (3)	0.005 (3)	−0.001 (2)
C1A	0.052 (3)	0.033 (3)	0.042 (3)	−0.004 (2)	0.000 (2)	0.000 (2)
C2A	0.041 (2)	0.037 (3)	0.043 (3)	0.000 (2)	0.002 (2)	0.000 (2)
C3A	0.053 (3)	0.038 (3)	0.052 (3)	−0.003 (2)	0.007 (3)	−0.004 (2)
C4A	0.057 (3)	0.036 (3)	0.045 (3)	0.003 (2)	−0.006 (2)	−0.002 (2)
C5A	0.063 (3)	0.052 (4)	0.081 (4)	0.007 (3)	0.017 (3)	−0.004 (3)
C6A	0.088 (4)	0.054 (4)	0.094 (5)	0.003 (3)	0.019 (4)	−0.022 (4)
C7A	0.085 (4)	0.042 (3)	0.084 (5)	−0.003 (3)	−0.006 (4)	−0.008 (3)
C8A	0.073 (4)	0.042 (3)	0.085 (5)	−0.015 (3)	0.008 (4)	−0.001 (3)
C9A	0.055 (3)	0.041 (3)	0.074 (4)	−0.005 (2)	0.004 (3)	−0.006 (3)
C10A	0.054 (3)	0.036 (3)	0.052 (3)	0.001 (2)	0.006 (3)	0.004 (2)
C11A	0.101 (3)	0.102 (3)	0.100 (3)	−0.0004 (10)	0.0069 (10)	0.0018 (10)
C12A	0.159 (4)	0.161 (4)	0.159 (4)	0.0003 (10)	0.0108 (11)	0.0007 (10)
C13A	0.075 (3)	0.036 (3)	0.049 (3)	−0.006 (2)	0.001 (3)	−0.004 (2)
C14A	0.045 (2)	0.039 (3)	0.043 (3)	0.006 (2)	−0.005 (2)	−0.002 (2)
C15A	0.042 (3)	0.040 (3)	0.056 (3)	−0.005 (2)	0.002 (2)	−0.008 (2)
C16A	0.052 (3)	0.052 (3)	0.071 (4)	0.000 (3)	−0.005 (3)	−0.019 (3)
C17A	0.049 (3)	0.056 (3)	0.050 (3)	0.009 (3)	−0.005 (2)	−0.005 (3)
C18A	0.044 (3)	0.062 (4)	0.066 (4)	−0.001 (3)	0.000 (3)	−0.008 (3)
C19A	0.044 (3)	0.046 (3)	0.056 (3)	−0.003 (2)	0.009 (2)	−0.006 (2)
Br1B	0.1217 (6)	0.0991 (6)	0.0771 (5)	0.0286 (5)	0.0117 (4)	0.0363 (4)
O1B	0.065 (2)	0.054 (2)	0.065 (2)	0.0025 (18)	0.022 (2)	0.0053 (19)
O2B	0.081 (3)	0.100 (3)	0.072 (3)	0.003 (3)	0.035 (2)	0.002 (2)
O3B	0.087 (3)	0.080 (3)	0.044 (2)	−0.019 (2)	0.0017 (19)	−0.015 (2)
O4B	0.067 (3)	0.109 (4)	0.096 (4)	0.022 (3)	0.020 (3)	0.006 (3)
O5B	0.160 (5)	0.103 (5)	0.167 (6)	0.050 (4)	−0.062 (5)	−0.078 (5)
N1B	0.094 (4)	0.070 (4)	0.052 (3)	0.025 (3)	−0.002 (3)	0.000 (3)
C1B	0.045 (3)	0.049 (3)	0.048 (3)	−0.004 (2)	0.004 (2)	−0.003 (2)
C2B	0.046 (3)	0.045 (3)	0.044 (3)	−0.005 (2)	0.007 (2)	0.002 (2)
C3B	0.046 (3)	0.044 (3)	0.051 (3)	−0.003 (2)	0.008 (2)	0.001 (2)
C4B	0.049 (3)	0.050 (3)	0.050 (3)	−0.002 (2)	0.003 (2)	−0.005 (3)
C5B	0.060 (3)	0.058 (4)	0.049 (3)	0.007 (3)	0.007 (3)	0.009 (3)
C6B	0.076 (4)	0.066 (4)	0.059 (4)	0.001 (3)	−0.008 (3)	0.004 (3)
C7B	0.059 (3)	0.052 (4)	0.073 (4)	0.006 (3)	0.003 (3)	0.000 (3)
C8B	0.058 (3)	0.061 (4)	0.083 (4)	−0.002 (3)	0.012 (3)	−0.002 (3)
C9B	0.051 (3)	0.056 (4)	0.065 (4)	−0.001 (3)	0.007 (3)	0.002 (3)
C10B	0.060 (3)	0.055 (3)	0.052 (3)	0.004 (3)	0.009 (3)	0.005 (3)
C11B	0.105 (3)	0.105 (3)	0.104 (3)	−0.0009 (10)	0.0065 (10)	−0.0009 (10)
C12B	0.133 (3)	0.133 (3)	0.132 (3)	−0.0001 (10)	0.0087 (10)	−0.0007 (10)
C13B	0.061 (3)	0.059 (3)	0.050 (3)	0.009 (3)	0.003 (2)	−0.004 (3)
C14B	0.039 (2)	0.050 (3)	0.047 (3)	−0.002 (3)	−0.002 (2)	0.001 (3)
C15B	0.048 (3)	0.070 (4)	0.059 (3)	−0.011 (3)	−0.002 (3)	0.005 (3)
C16B	0.053 (3)	0.085 (5)	0.054 (4)	0.010 (3)	−0.013 (3)	0.011 (3)
C17B	0.067 (4)	0.064 (4)	0.052 (3)	0.015 (3)	0.007 (3)	−0.009 (3)
C18B	0.067 (3)	0.050 (3)	0.059 (4)	−0.011 (3)	0.009 (3)	0.004 (3)
C19B	0.051 (3)	0.061 (3)	0.050 (3)	−0.004 (3)	−0.005 (2)	0.006 (3)

### Geometric parameters (Å, °)

**Table:**

Br1A—C17A	1.872 (5)	Br1B—C17B	1.899 (6)
O1A—C3A	1.221 (6)	O1B—C3B	1.212 (5)
O2A—C10A	1.197 (6)	O2B—C10B	1.201 (6)
O3A—C10A	1.324 (6)	O3B—C10B	1.328 (7)
O3A—C11A	1.420 (9)	O3B—C11B	1.470 (9)
O4A—N1A	1.201 (6)	O4B—N1B	1.219 (7)
O5A—N1A	1.222 (6)	O5B—N1B	1.193 (7)
N1A—C13A	1.490 (7)	N1B—C13B	1.505 (7)
C1A—C13A	1.522 (7)	C1B—C14B	1.510 (7)
C1A—C14A	1.524 (6)	C1B—C13B	1.533 (7)
C1A—C2A	1.542 (6)	C1B—C2B	1.568 (7)
C1A—H1A	0.9800	C1B—H1B	0.9800
C2A—C10A	1.513 (7)	C2B—C10B	1.518 (7)
C2A—C3A	1.536 (7)	C2B—C3B	1.530 (7)
C2A—H2A	0.9800	C2B—H2B	0.9800
C3A—C4A	1.497 (7)	C3B—C4B	1.486 (7)
C4A—C5A	1.377 (7)	C4B—C5B	1.392 (7)
C4A—C9A	1.403 (7)	C4B—C9B	1.395 (7)
C5A—C6A	1.382 (8)	C5B—C6B	1.380 (8)
C5A—H5A	0.9300	C5B—H5B	0.9300
C6A—C7A	1.379 (9)	C6B—C7B	1.379 (8)
C6A—H6A	0.9300	C6B—H6B	0.9300
C7A—C8A	1.363 (8)	C7B—C8B	1.375 (8)
C7A—H7A	0.9300	C7B—H7B	0.9300
C8A—C9A	1.371 (7)	C8B—C9B	1.376 (8)
C8A—H8A	0.9300	C8B—H8B	0.9300
C9A—H9A	0.9300	C9B—H9B	0.9300
C11A—C12A	1.527 (3)	C11B—C12B	1.528 (3)
C11A—H11A	0.9700	C11B—H11C	0.9700
C11A—H11B	0.9700	C11B—H11D	0.9700
C12A—H12A	0.9600	C12B—H12D	0.9600
C12A—H12B	0.9600	C12B—H12E	0.9600
C12A—H12C	0.9600	C12B—H12F	0.9600
C13A—H13A	0.9700	C13B—H13C	0.9700
C13A—H13B	0.9700	C13B—H13D	0.9700
C14A—C15A	1.384 (7)	C14B—C15B	1.382 (7)
C14A—C19A	1.387 (7)	C14B—C19B	1.386 (7)
C15A—C16A	1.382 (7)	C15B—C16B	1.393 (8)
C15A—H15A	0.9300	C15B—H15B	0.9300
C16A—C17A	1.387 (7)	C16B—C17B	1.349 (8)
C16A—H16A	0.9300	C16B—H16B	0.9300
C17A—C18A	1.389 (7)	C17B—C18B	1.387 (8)
C18A—C19A	1.385 (7)	C18B—C19B	1.390 (7)
C18A—H18A	0.9300	C18B—H18B	0.9300
C19A—H19A	0.9300	C19B—H19B	0.9300
			
C10A—O3A—C11A	117.1 (5)	C10B—O3B—C11B	117.2 (5)
O4A—N1A—O5A	123.3 (5)	O5B—N1B—O4B	124.2 (6)
O4A—N1A—C13A	118.9 (5)	O5B—N1B—C13B	117.1 (6)
O5A—N1A—C13A	117.7 (5)	O4B—N1B—C13B	118.7 (6)
C13A—C1A—C14A	112.3 (4)	C14B—C1B—C13B	110.9 (4)
C13A—C1A—C2A	107.1 (4)	C14B—C1B—C2B	112.9 (4)
C14A—C1A—C2A	112.8 (4)	C13B—C1B—C2B	109.1 (4)
C13A—C1A—H1A	108.2	C14B—C1B—H1B	107.9
C14A—C1A—H1A	108.2	C13B—C1B—H1B	107.9
C2A—C1A—H1A	108.2	C2B—C1B—H1B	107.9
C10A—C2A—C3A	107.6 (4)	C10B—C2B—C3B	110.3 (4)
C10A—C2A—C1A	110.8 (4)	C10B—C2B—C1B	108.5 (4)
C3A—C2A—C1A	110.7 (4)	C3B—C2B—C1B	112.3 (4)
C10A—C2A—H2A	109.2	C10B—C2B—H2B	108.5
C3A—C2A—H2A	109.2	C3B—C2B—H2B	108.5
C1A—C2A—H2A	109.2	C1B—C2B—H2B	108.5
O1A—C3A—C4A	120.5 (5)	O1B—C3B—C4B	120.6 (4)
O1A—C3A—C2A	119.5 (4)	O1B—C3B—C2B	119.9 (4)
C4A—C3A—C2A	119.9 (4)	C4B—C3B—C2B	119.6 (4)
C5A—C4A—C9A	119.5 (5)	C5B—C4B—C9B	118.4 (5)
C5A—C4A—C3A	117.7 (5)	C5B—C4B—C3B	118.7 (4)
C9A—C4A—C3A	122.8 (5)	C9B—C4B—C3B	122.8 (5)
C4A—C5A—C6A	120.6 (6)	C6B—C5B—C4B	120.3 (5)
C4A—C5A—H5A	119.7	C6B—C5B—H5B	119.8
C6A—C5A—H5A	119.7	C4B—C5B—H5B	119.8
C7A—C6A—C5A	119.1 (6)	C7B—C6B—C5B	120.7 (6)
C7A—C6A—H6A	120.4	C7B—C6B—H6B	119.7
C5A—C6A—H6A	120.4	C5B—C6B—H6B	119.7
C8A—C7A—C6A	120.8 (6)	C6B—C7B—C8B	119.4 (5)
C8A—C7A—H7A	119.6	C6B—C7B—H7B	120.3
C6A—C7A—H7A	119.6	C8B—C7B—H7B	120.3
C7A—C8A—C9A	120.8 (6)	C9B—C8B—C7B	120.6 (5)
C7A—C8A—H8A	119.6	C9B—C8B—H8B	119.7
C9A—C8A—H8A	119.6	C7B—C8B—H8B	119.7
C8A—C9A—C4A	119.2 (5)	C8B—C9B—C4B	120.6 (5)
C8A—C9A—H9A	120.4	C8B—C9B—H9B	119.7
C4A—C9A—H9A	120.4	C4B—C9B—H9B	119.7
O2A—C10A—O3A	124.9 (5)	O2B—C10B—O3B	124.7 (5)
O2A—C10A—C2A	124.2 (5)	O2B—C10B—C2B	125.0 (5)
O3A—C10A—C2A	110.9 (4)	O3B—C10B—C2B	110.3 (4)
O3A—C11A—C12A	111.1 (7)	O3B—C11B—C12B	110.5 (7)
O3A—C11A—H11A	109.4	O3B—C11B—H11C	109.5
C12A—C11A—H11A	109.4	C12B—C11B—H11C	109.5
O3A—C11A—H11B	109.4	O3B—C11B—H11D	109.5
C12A—C11A—H11B	109.4	C12B—C11B—H11D	109.5
H11A—C11A—H11B	108.0	H11C—C11B—H11D	108.1
C11A—C12A—H12A	109.5	C11B—C12B—H12D	109.5
C11A—C12A—H12B	109.5	C11B—C12B—H12E	109.5
H12A—C12A—H12B	109.5	H12D—C12B—H12E	109.5
C11A—C12A—H12C	109.5	C11B—C12B—H12F	109.5
H12A—C12A—H12C	109.5	H12D—C12B—H12F	109.5
H12B—C12A—H12C	109.5	H12E—C12B—H12F	109.5
N1A—C13A—C1A	113.5 (4)	N1B—C13B—C1B	110.0 (4)
N1A—C13A—H13A	108.9	N1B—C13B—H13C	109.7
C1A—C13A—H13A	108.9	C1B—C13B—H13C	109.7
N1A—C13A—H13B	108.9	N1B—C13B—H13D	109.7
C1A—C13A—H13B	108.9	C1B—C13B—H13D	109.7
H13A—C13A—H13B	107.7	H13C—C13B—H13D	108.2
C15A—C14A—C19A	118.1 (4)	C15B—C14B—C19B	117.8 (5)
C15A—C14A—C1A	120.1 (4)	C15B—C14B—C1B	119.6 (4)
C19A—C14A—C1A	121.8 (4)	C19B—C14B—C1B	122.5 (4)
C16A—C15A—C14A	122.3 (5)	C14B—C15B—C16B	121.4 (5)
C16A—C15A—H15A	118.8	C14B—C15B—H15B	119.3
C14A—C15A—H15A	118.8	C16B—C15B—H15B	119.3
C15A—C16A—C17A	119.4 (5)	C17B—C16B—C15B	119.3 (5)
C15A—C16A—H16A	120.3	C17B—C16B—H16B	120.3
C17A—C16A—H16A	120.3	C15B—C16B—H16B	120.3
C16A—C17A—C18A	118.7 (5)	C16B—C17B—C18B	121.6 (5)
C16A—C17A—Br1A	120.2 (4)	C16B—C17B—Br1B	119.6 (4)
C18A—C17A—Br1A	121.0 (4)	C18B—C17B—Br1B	118.8 (5)
C19A—C18A—C17A	121.4 (5)	C17B—C18B—C19B	118.3 (5)
C19A—C18A—H18A	119.3	C17B—C18B—H18B	120.8
C17A—C18A—H18A	119.3	C19B—C18B—H18B	120.8
C18A—C19A—C14A	120.1 (5)	C14B—C19B—C18B	121.5 (5)
C18A—C19A—H19A	120.0	C14B—C19B—H19B	119.2
C14A—C19A—H19A	120.0	C18B—C19B—H19B	119.2
			
C13A—C1A—C2A—C10A	169.2 (4)	C14B—C1B—C2B—C10B	41.7 (5)
C14A—C1A—C2A—C10A	45.2 (5)	C13B—C1B—C2B—C10B	165.5 (4)
C13A—C1A—C2A—C3A	−71.5 (5)	C14B—C1B—C2B—C3B	164.0 (4)
C14A—C1A—C2A—C3A	164.5 (4)	C13B—C1B—C2B—C3B	−72.2 (5)
C10A—C2A—C3A—O1A	92.0 (6)	C10B—C2B—C3B—O1B	121.8 (5)
C1A—C2A—C3A—O1A	−29.2 (7)	C1B—C2B—C3B—O1B	0.6 (7)
C10A—C2A—C3A—C4A	−85.8 (6)	C10B—C2B—C3B—C4B	−57.3 (6)
C1A—C2A—C3A—C4A	153.0 (4)	C1B—C2B—C3B—C4B	−178.5 (4)
O1A—C3A—C4A—C5A	7.5 (8)	O1B—C3B—C4B—C5B	−7.0 (7)
C2A—C3A—C4A—C5A	−174.6 (5)	C2B—C3B—C4B—C5B	172.1 (5)
O1A—C3A—C4A—C9A	−173.9 (5)	O1B—C3B—C4B—C9B	173.3 (5)
C2A—C3A—C4A—C9A	3.9 (8)	C2B—C3B—C4B—C9B	−7.6 (7)
C9A—C4A—C5A—C6A	2.9 (9)	C9B—C4B—C5B—C6B	0.6 (8)
C3A—C4A—C5A—C6A	−178.4 (6)	C3B—C4B—C5B—C6B	−179.1 (5)
C4A—C5A—C6A—C7A	−2.3 (10)	C4B—C5B—C6B—C7B	−0.9 (9)
C5A—C6A—C7A—C8A	1.8 (10)	C5B—C6B—C7B—C8B	1.3 (9)
C6A—C7A—C8A—C9A	−2.1 (10)	C6B—C7B—C8B—C9B	−1.5 (9)
C7A—C8A—C9A—C4A	2.8 (9)	C7B—C8B—C9B—C4B	1.3 (9)
C5A—C4A—C9A—C8A	−3.2 (9)	C5B—C4B—C9B—C8B	−0.9 (8)
C3A—C4A—C9A—C8A	178.3 (5)	C3B—C4B—C9B—C8B	178.9 (5)
C11A—O3A—C10A—O2A	4.1 (8)	C11B—O3B—C10B—O2B	−2.7 (8)
C11A—O3A—C10A—C2A	−176.0 (5)	C11B—O3B—C10B—C2B	177.7 (5)
C3A—C2A—C10A—O2A	−71.6 (6)	C3B—C2B—C10B—O2B	125.2 (6)
C1A—C2A—C10A—O2A	49.5 (7)	C1B—C2B—C10B—O2B	−111.3 (6)
C3A—C2A—C10A—O3A	108.4 (5)	C3B—C2B—C10B—O3B	−55.2 (6)
C1A—C2A—C10A—O3A	−130.5 (4)	C1B—C2B—C10B—O3B	68.3 (5)
C10A—O3A—C11A—C12A	−132.7 (7)	C10B—O3B—C11B—C12B	93.6 (7)
O4A—N1A—C13A—C1A	−34.8 (7)	O5B—N1B—C13B—C1B	141.9 (6)
O5A—N1A—C13A—C1A	148.1 (5)	O4B—N1B—C13B—C1B	−37.6 (7)
C14A—C1A—C13A—N1A	−62.7 (6)	C14B—C1B—C13B—N1B	−56.3 (5)
C2A—C1A—C13A—N1A	173.0 (4)	C2B—C1B—C13B—N1B	178.8 (4)
C13A—C1A—C14A—C15A	97.8 (5)	C13B—C1B—C14B—C15B	122.3 (5)
C2A—C1A—C14A—C15A	−141.2 (5)	C2B—C1B—C14B—C15B	−114.9 (5)
C13A—C1A—C14A—C19A	−81.7 (6)	C13B—C1B—C14B—C19B	−54.4 (6)
C2A—C1A—C14A—C19A	39.4 (6)	C2B—C1B—C14B—C19B	68.4 (6)
C19A—C14A—C15A—C16A	0.4 (7)	C19B—C14B—C15B—C16B	1.1 (8)
C1A—C14A—C15A—C16A	−179.0 (5)	C1B—C14B—C15B—C16B	−175.7 (5)
C14A—C15A—C16A—C17A	−0.6 (8)	C14B—C15B—C16B—C17B	−0.1 (9)
C15A—C16A—C17A—C18A	0.5 (8)	C15B—C16B—C17B—C18B	−1.1 (9)
C15A—C16A—C17A—Br1A	177.5 (4)	C15B—C16B—C17B—Br1B	177.4 (4)
C16A—C17A—C18A—C19A	−0.2 (8)	C16B—C17B—C18B—C19B	1.3 (8)
Br1A—C17A—C18A—C19A	−177.2 (4)	Br1B—C17B—C18B—C19B	−177.2 (4)
C17A—C18A—C19A—C14A	0.0 (8)	C15B—C14B—C19B—C18B	−0.9 (8)
C15A—C14A—C19A—C18A	−0.2 (7)	C1B—C14B—C19B—C18B	175.8 (5)
C1A—C14A—C19A—C18A	179.3 (5)	C17B—C18B—C19B—C14B	−0.3 (8)
